# Interprofessional analysis of esthetical deformity from long head biceps tenotomy

**DOI:** 10.1590/1413-78522015230300898

**Published:** 2015

**Authors:** Alexandre Almeida, Márcio Rangel Valin, Cleber Lotti, Nayvaldo Couto de Almeida, Ana Paula Agostini

**Affiliations:** 1 Hospital Saúde de Caixas do Sul, Caxias do Sul, RS, Brasil; 2 Hospital Pompéia, Caxias do Sul, RS, Brasil; 3 Pontifícia Universidade Católica do Rio Grande do Sul, Porto Alegre, RS, Brasil

**Keywords:** Shoulder/surgery, Arthroscopy, Tenotomy, Obesity

## Abstract

**OBJECTIVE:**

To evaluate the perception of an esthetical deformity resultant from arthroscopic long head biceps (LHB) tenotomy according to the degree of experience of the assisting professional.

**METHODS:**

120 patients submitted to shoulder surgery were photographed and photos were mounted in a PowerPoint presentation. Three shoulder specialist surgeons, three generalist orthopedic surgeons and three graduated residents analyzed the presentation.

**RESULTS:**

On all patients we observed most agreement among the shoulder specialists. When just the patients with LHB tenotomy were analyzed, the specialists agreed moderately, the generalists had small agreement and the residents, a poor one. Analyzing patients with BMI < 30, there was major agreement between the specialists, while the generalists and residents had poor agreement. Analyzing patients with BMI ≥ 30, the generalists had small kappa agreement, while the specialists and residents had no agreement.

**CONCLUSIONS:**

The perception of an esthetical deformity regarding a LHB tenotomy did not have significant agreement between different level of professionals, even though the specialists showed similar perception on tenotomy patients. The evaluation of obese patients lowered the agreement on the three groups of professionals. *Level of Evidence III. Case Control Study.*

## INTRODUCTION

The simple tenotomy of a compromised long head of the biceps (LHB), whether held in isolation or associated with other procedures is an approach recognized to improve postoperative outcomes of patients submitted to various therapeutic procedures in the shoulder.^1^^-^ 14 Its criterion is well defined in the literature: it is indicated for injuries that compromise 50% or more of the thickness of the tendon to the instability in the bicipital groove or for a degenerative SLAP injury in elderly patients.^4^^,^ 5 ^,^ 15 ^-^ 18

The tenotomy may lead to the emergence of an aesthetic deformity and a painful muscle spasm resulting of distal migration of the CLB under the effect of muscle traction.^19^ This approach divides surgeons between those who perform tenotomy with confidence and those who express much concern regarding the aesthetic consequences of this procedure.

The perception of the aesthetic deformity is evaluated in the literature very confusely.^20^ Some authors report the perception of the deformity as a personal evaluation of their patients. Walch et al.^8^ suggest that some peculiarities such as poor muscle tone in elderly patients can mask the deformity.

Most studies question patients about the perception or not of aesthetic deformity resulting from tenotomy of LHB, these rates vary between 5 and 65%.^8^^,^ 13 ^,^ 21 ^-^ 28 Almeida et al.^22^ showed that the perception of aesthetic deformity by patient was higher in men with the operated dominant upper limb (UL), BMI < 30 kg/m^2^, abdominal skinfold < 23.2 mm and contralateral tricipital fold < 14.5 mm. Godinho et al.^28^ stated that the assessment of cosmetic deformity is more concise when performed by an professional orthopedist.

The objective of this research is to analyze comparatively whether the perception of the aesthetic deformity resulting of tenotomy of LHB varies according to the degree of specialization of the assisting professional.

## METHODS

This is a cross-sectional study. A group of 120 patients who underwent arthroscopic surgery on a shoulder in the period from June 12, 2002 and December 3, 2008 was analyzed. The study was approved by the Institutional Ethics Committee.

There were not selected to participate in this study patients with atrophy or aesthetic modification of the contralateral upper limb, compromising the comparison between the upper limbs such as the finding of a breach of contralateral LHB; history of fractures and/or surgery in the contralateral upper limb (UL).

The mean age of the 120 patients included in the study was 53.9 ± 11.9 years old. Regarding gender, 80 patients were female (66.7%) and 40 patients were male. The dominant side was affected in 93 patients (73.5%).

Within this universe of 120 patients, tenotomy of the LHB was performed in 69 patients. The average age of this group was 58.7 ± 10.4 years old. Regarding gender, 50 patients were female (72.5%) and 19 male.

The other 51 patients who underwent arthroscopic shoulder surgery without tenotomy of the LHB had a mean age of 47.5 ± 10.9 years. Regarding gender, 30 patients were female (58.8%) and 21 male.

All patients underwent measurement of height in centimeters and weight in kilograms in the immediate preoperative period. The values ​​were used to calculate the Body Mass Index (BMI) by the specific equation.^29^ The result is obtained by dividing the weight (in kilograms) by the square of the height (in meters). Its result is given in kg/m². (BMI = weight/height^2^)

Patients were classified according to BMI between different degrees of obesity according to Table 1.^29^

**Table 1.** Obesity degree and obesity rating.

BMI (kg/m2)	
18 - 24.9	Normal
25 - 29.9	Overweight
30 - 34.9	Obesity G1
35 - 39.9	Obesity G2
>= 40.0	Morbid Obesity

BMI: Body mass index in Kg/m2.

After anesthesia, patients were placed in the lateral position with the upper limb at 30° flexed at 20° and under traction of 5 Kg. Tenotomy of LHB was performed with a Trimmer clamp at its insertion on the upper lip of the glenoid when: 50% or more of the tendon thickness was impaired, instability in the intertubercular groove was diagnosed or a degenerative SLAP was found. The procedure was always performed by the same surgeon.

All patients still anesthetized were immobilized in the operating room with a sling. An abduction cushion was used when the injury of the rotator cuff sutured was large or extensive.

Patients who underwent tenotomy of LHB were instructed to avoid forced flexion of the elbow, as well as its entire length within the first four weeks after surgery.

All 120 patients in the study were evaluated with a median of 16.5 months (IIQ 8 to 23.8) postoperatively. During the evaluation, all patients received the Informed Consent form approved by the Ethics Committee of the institution where the work was performed.

Patients were photographed with a digital camera Sony Cybershot W-100 in VGA resolution at a standard distance of 60 cm, with the UL aducted by the trunk, the elbow at 90 degrees and maximum supination. A flash light was used regardless of the room light conditions. The photos showed the arm with the adjacent shoulder and elbow joints. The patient's face was hidden.

**Figure 1.**
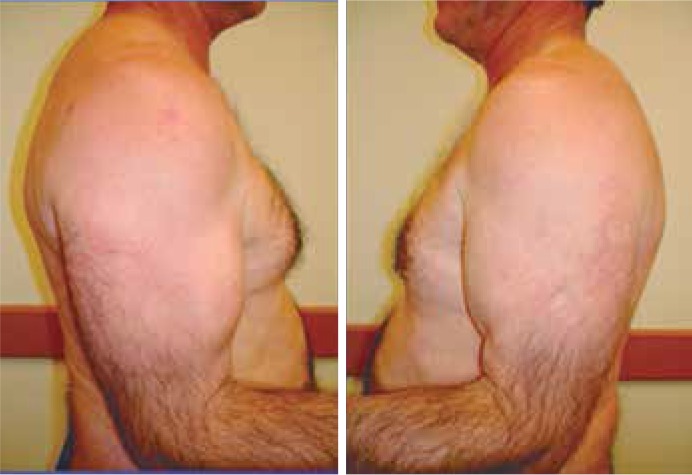
Patient tenotomized on its right shoulder

**Figure 2.**
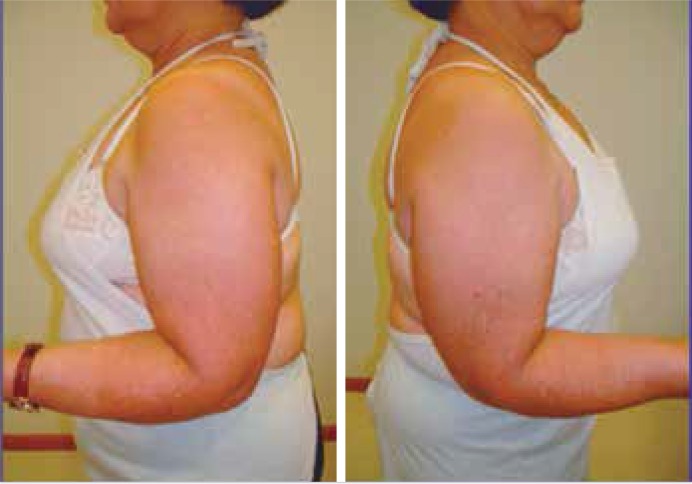
Patient tenotomized on its left shoulder.

**Figure 3.**
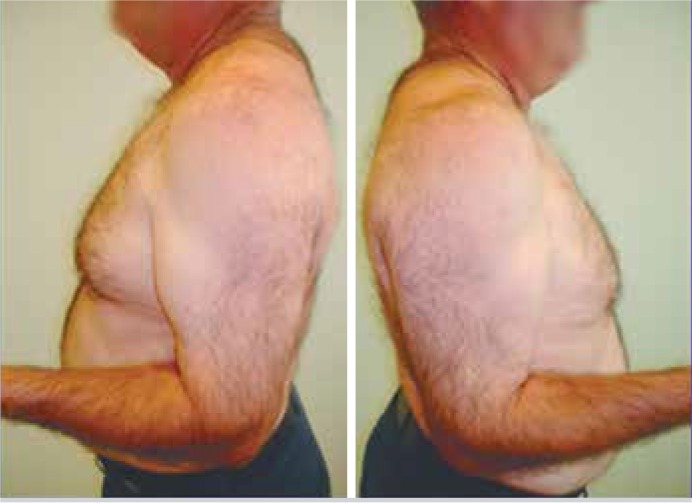
Patient tenotomized on its left shoulder.

The photos of the patients were placed in a Microsoft PowerPoint (PowerPoint version 12.1.2-2008 for Mac) presentation with a blue background and size of 8 x 5 cm. Photos of the UL operated were on the left and the photo of the contralateral limb on the right side, for comparison. No description of the patient data or clinical history was revealed. (Figures 1, 2 and 3)

The Microsoft PowerPoint presentation was submitted for assessment by three professionals specialized in shoulder surgery, three generalist orthopedic professionals and three orthopedics resident doctors from the third year on. The professionals were invited to observe each slide separately for 60 sec at most and score the response grid in case they observed or not any aesthetic deformity that could be the result of a tenotomy of the LHB.

The variables studied were age, gender, operated side, dominance, degree of obesity and the correlation between opinions of the medical professionals.

Data were analyzed with SPSS (Statistical Package for Social Sciences) version 12.0 (SPSS Inc. 1989-2003). For statistical analysis, we used: calculation of mean, standard deviation, median, frequency and percentage. We used the Student *t*-test for age assessment. The chi-square test was used to evaluate the side, gender, dominance and BMI. Differences with p ≤ 0.05 for a 95% confidence interval were considered significant.

The Kappa index was used to assess agreement between the observations of different professionals. It is a measure of interobserver agreement and measures the degree of agreement beyond what would be expected solely by chance. This measurement of agreement has as maximum value one, representing total agreement and values ​​close to and below zero, indicating no agreement, or even that the agreement was exactly as expected by chance. A possible Kappa value lower than zero (negative), suggests that the correlation found was less than that expected solely by chance. (Table 2)

**Table 2.** Kappa Index.

Kappa values	Interpretation
<0	No agreement
0-0.19	Poor
0.20-0.39	Low
0.40-0.59	Moderate
0.60-0.79	Substantial
0.80-1.00	Almost perfect

## RESULTS

The study evaluated a total of 120 patients. There was no statistically significant difference between the groups of patients undergoing shoulder arthroscopy with or without tenotomy of the LHB when the following variables were evaluated: mean age (p=0.847), gender (p=0.117), operated side (p=0.042) , dominance (p=0.119) and BMI (p=0.631). The groups were considered similar. (Table 3)

**Table 3.** Description of groups.

	Tenotomy n=69	No tenotomy n=51	P
Age (years old) mean (sd)	58.7 (10.4)	47.5 (10.9)	0.847*
Gender (Feminine) n (%)	50 (72.5)	30 (58.8)	0.117**
Side (Right) n (%)	56 (81.2)	33 (64.7)	0.042**
Dominant side n (%)	57 (82.6)	36 (70.6)	0.119**
BMI ≥ 30 kg/m^2 ^n (%)	15 (21.7)	13 (25.5)	0.631**

* Student t-test;

** Chi-square test; sd: Standard deviation.

When analyzing the overall sample of patients (n=120) we observed a greater agreement among orthopedic surgeons specialists in shoulder surgery (Kappa 0.49 (range 0.38 to 0.59) / p<0.001). The general orthopedists (Kappa 0.29 (range 0.19 to 0.39) / p<0.001) and resident physicians (Kappa 0.23 (range 0.12 to 0.33) / p<0.001) had a small agreement. (Table 4)

**Table 4.** General assessment of agreement. (N=120).

Observers		% who noticed deformity		% Agreement	Kappa (CI 95%)	P	Overall Kappa (CI 95%)
Shoulder specialist	1 x 2	23.3	14.2	82.5	0.43 (0.24-0.63)	< 0.001	0.49 (0.38-0.59) P<0.001
	1 x 3	23.3	13.3	86.7	0.56 (0.38-0.75)	<0.001	
	2 x 3	14.2	13.3	87.5	0.47 (0.25-0.70)	< 0.001	
General Orthopedist	1 x 2	40.8	45.8	66.7	0.32 (0.15-0.49)	<0.001	0.29 (0.19-0.39) P<0.001
	1 x 3	40.8	45.0	55.8	0.10 (-0.08-0.28)	0.271	
	2 x 3	45.8	45.0	72.5	0.45 (0.28-0.61)	< 0.001	
Resident	1 x 2	35.0	15.8	69.2	0.22 (0.06-0.39)	0.005	0.23 (0.12-0.33) P<0.001
	1 x 3	35.0	35.0	70.0	0.34 (0.17-0.52)	<0.001	
	2X3	15.8	35.0	65.8	0.14 (-0.03-0.31)	0.079	

Kappa index: Agreement assessment.

When we analyzed the sample of patients who underwent tenotomy of the LHB, the experts agreed with each other surgeons moderately (Kappa 0.51 (range 0.38 to 0.65) / p <0.001). However, this correlation does not appear to be statistically different from the general orthopedists, having a small correlation (Kappa 0.30 (range 0.17 to 0.44) / p <0.001). The resident doctors had a poor agreement (Kappa 0.17 (range 0.04 to 0.31) / p=0.013), but statistically it is not very different from the general orthopedists.

When we analyzed the sample of patients which did not undergo tenotomy of the LHB, poor agreement was observed among surgeon specialists (Kappa 0.17 (range 0.02 to 0.33) / p=0.032) and small among the general orthopedists (Kappa 0.21 (range 0.05 to 0.36) / p=0.011) and resident physicians (Kappa 0.21 (range 0.05 to 0.37) / p=0.009).

When we analyzed the sample of patients with BMI <30 kg/m^2^ there was a moderate to substantial agreement among expert surgeons (Kappa 0.54 (range 0.42 to 0.66) / p <0.001), while the general orthopedists (Kappa 0.28 (range 0.16 to 0.40) / p <0.001) and resident physicians (Kappa 0.24 (range 0.12 to 0.36) / p <0.001) remained with poor agreement. (Table 5)

**Table 5.** Assessment of agreement in patients with BMI < 30 kg/m2 (N=92).

Observers		% who noticed deformity		% Agreement	Kappa (CI 95%)	P	Overall Kappa (CI 95%)
Shoulder specialist	1 x 2	26.1	17.4	82.6	0.50 (0.29-0.70)	< 0.001	0.54 (0.42-0.66) P < 0.001
	1 x 3	26.1	15.2	86.9	0.61 (0.42-0.80)	< 0.001	
	2 x 3	17.4	15.2	87.0	0.52 (0.29-0.76)	< 0.001	
General Orthopedist	1 x 2	43.5	45.7	65.2	0.30 (0.10-0.49)	0.004	0.28 (0.16-0.40) P < 0.001
	1 x 3	43.5	45.7	56.5	0.12 (-0.08-0.32)	0.247	
	2 x 3	45.7	45.7	71.7	0.43 (0.25-0.61)	< 0.001	
Resident	1 x 2	34.8	19.6	71.7	0.31 (0.11-0.50)	0.002	0.24 (0.12-0.36) P < 0.001
	1 x 3	34.8	39.1	69.5	0.35 (0.15-0.54)	0.001	
	2 x 3	19.6	39.1	60.9	0.10 (-0.09-0.29)	0.292	

Kappa index: Agreement assessment.

When we analyzed the sample of patients with BMI ≥ 30 kg/m^2^ general orthopedists had low agreement (Kappa 0.31 (range 0.09 to 0.52) / p=0.005). For experts surgeons (Kappa 0.07 (range -0.15-0.28) / p=0.552) and resident physicians (Kappa 0.12 (range -0.10-0.33) / p=0.292) there wasn't any agreement. (Table 6)

**Table 6.** Assessment of agreement in patients with BMI > 30 kg/m2 (N=28).

Observers		% who noticed deformity		% Agreement	Kappa (CI 95%)	P	Overall Kappa (CI 95%)
Shoulder specialist	1 x 2	14.3	3.6	82.1	-0.06 (-0.16-0.04)	0.678	0.07 (-015-0.28) P = 0.552
	1 x 3	14.3	7.1	85.7	0.26 (-0.24-0.77)	0.134	
	2 x 3	3.6	7.1	89.3	-0.05 (-0.12-0.02)	0.778	
General Orthopedist	1 x 2	32.1	46.4	71.4	0.41 (0.09-0.74)	0.022	0.31 (0.09-0.52) P = 0.005
	1 x 3	32.1	42.9	53.6	0.02 (-0.34-0.38)	0.907	
	2 x 3	46.4	42.9	75.0	0.50 (0.17-0.82)	0.09	
Resident	1 x 2	35.7	3.6	60.7	-0.07 (-020-0.06)	0.448	0.12 (-0.10-0.33) P = 0.292
	1 x 3	35.7	21.4	71.4	0.32 (-0.04-0.68)	0.074	
		3.6	21.4	82.2	0.24 (-0.15-0.63)	0.051	

Kappa index: Agreement assessment.

## DISCUSSION

The literature lacks a definition of the frequency of aesthetic deformity following the distal migration of the LHB after performing tenotomy.^19^^,^ 20

Most authors questioned patients about their perception of the aesthetic aspect of the UL. Boileau et al.^21^ reported that their patients perceived the deformity in 66.6% of cases where isolated tenotomy of LHB was performed, without clinical or aesthetic significance. Almeida et al.^22^ found aesthetic complaint in 35.1% of the sample. Maynou et al.^13^ noticed complaint to the asymmetry of the UL in only 5% of their patients. Veado et al.^30^ showed no complaints regarding aesthetic deformity of UL in LHB tenotomized patients with irreparable injuries of the UL. The perception and concern about aesthetic deformities may vary according to socioeconomic, environmental, cultural, and professional and physical activities. Duff and Campbell^23^ compared the results of isolated tenotomy of LHB in young and active population with elderly and sedentary patients. They found no statistical difference between the groups. They found only 3% of patients concerned with their aesthetic deformity, none of which requested surgical repair.

Some authors have described their own perception of aesthetic deformity observing the postoperative period of their patients. Walch et al.^8 ^followed the result of 307 tenotomies the LHB and verified the presence of obvious aesthetic deformity in 50.2%. Kelly et al.^9 ^reported the presence of the Popeye sign at rest and during active elbow flexion in 70% of patients who underwent tenotomy of the LHB. Lim et al.^24^ found 45% while Delle Rose et al.^25^ verified the presence of Popeye sign on 37.5% of their tenotomized patients. De Carli et al.^26^ found 17% of deformity for the same procedure. Checchia et al.^31^ found only one case (8.3%) of a Popeye deformity in their series after performing tenotomy of the LHB for treating patients with irreparable UL injuries.

Godinho et al.^28^ demonstrated in a statistically significant way that the ability to verify the residual Popeye deformity is more concise in the professional physician. They used an independent examiner who had not participated in the surgical procedure to evaluate the presence of Popeye deformity after performing the LHB tenotomy technique associated with "jelly roll" tenodesis. They verified the aesthetic deformity in 31.8% of patients. In our study, there were available for examiners blindly, patients in postoperative of shoulder arthroscopy where tenotomy of the LHB could or could not have been performed. This fact may have been responsible for the difficulty encountered by the professionals in verifying the Popeye deformity.

Slenker et al. 27 performed a systematic review of the literature encompassing sixteen English-language articles comparing clinical outcomes among patients which underwent tenotomy of LHB and patients where the tenotomy was followed by tenodesis. The authors observed that the presence of aesthetic deformity occurred on average in 43% of patients with isolated tenotomy of the LHB.

The lack of standardization of questions and a way to assess aesthetic deformity leads to rates varying between 5 and 66% for determination of the presence of residual deformity that usually follows tenotomy of the LHB.^13^^,^ 21 ^-^ 27

The evaluation of the aesthetic deformity by the professional who performed the surgery creates an observation bias and may lead to overestimation of the findings. Similarly the more trained professional look with expertise in shoulder surgery could lead to more frequent perception of the deformity. We did not find in the literature studies with concern about the fact.

In our study (n = 120), we found a greater agreement among shoulder specialist orthopedic surgeons in perceiving patients with residual deformity from a LHB tenotomy, suggesting that the trained eye contributes to the situation. Among the general orthopedists and medical residents, the correlation was poor.

Walch et al.^8^ reported the difficulty in assessing the presence of deformity in obese or elderly patients with poor muscle tone, eventually classifying them as doubtful. Almeida et al.,^22^ also observed in a statistically significantly manner (p = 0.005) that obese patients perceive less the aesthetic deformity.

In our study, when we analyzed lean patients (BMI < 30 kg/m^2^) we saw an increase in agreement (moderate to substantial) among experts in shoulder surgery, while the general orthopedists and medical residents remained with poor agreement. This fact has further confirmed that the trained eye is better able to notice the aesthetic deformity.

Analysis of obese patients (BMI ≥ 30 kg/m^2^) lowered the level of agreement among the three professional classes, which was low among shoulder surgery specialists and total absent among the general orthopedists and medical residents. It is likely that little agreement among experts has been, in this study, an incidental finding, with no statistically significant difference of the divergence found in other professionals. We agree with Godinho et al.^28^ who suggest a correlation between the sensitivity of the patient and the professional to detect the residual deformity after tenotomy of the LHB, however, even the specialized professionals have difficulty visualizing the deformity in obese patients. Our findings suggest that the aggregating tenodesis of LHB in obese patients could be unnecessary, since the tenodesis does not increase the clinical and functional results when compared to the isolated tenotomy of the LHB.^26^^,^ 27 ^,^ 32 ^,^ 33

## CONCLUSIONS

The perception of the resulting aesthetic deformity resulting from tenotomy of LHB showed no significant correlation between shoulder surgery professional specialists, general orthopedists and medical residents, although the specialists demonstrated a similar perception of deformity in tenotomized patients.

Specialist professionals have shown greater agreement when evaluating patients with BMI < 30 kg/m^2^. The evaluation of obese patients with BMI ≥ 30 kg/m^2^ showed no correlation between the three different groups of professionals.
